# A Case of Prostatic Abscess with Malignant Lymphoma Involving the Prostate

**DOI:** 10.1155/2014/965823

**Published:** 2014-11-06

**Authors:** Wataru Noguchi, Yoshihiro Inoue, Mana Fukushima

**Affiliations:** ^1^Department of Urology, Omachi Municipal General Hospital, 3130 Omachi, Omachi City, Nagano Prefecture 398-0002, Japan; ^2^Department of Laboratory Medicine, Omachi Municipal General Hospital, Nagano 398-0002, Japan

## Abstract

Here, we report a case of prostatic abscess probably due to malignant lymphoma of the prostate. An 82-year-old man was referred to our hospital with chief complaints of urinary frequency and discomfort on urination. Antibiotics were prescribed, but the symptoms remained and intermittent fever appeared. The patient was diagnosed with prostatic abscess by computed tomography (CT). Digital rectal examination (DRE) revealed soft prostate, and thick pus was milked out from the extrameatus by prostatic massage. For drainage, we performed transurethral resection of the prostate (TURP). Drainage by TURP was successful as CT clearly showed reduction of prostatic abscess after the operation. Nevertheless, intermittent fever did not improve and the patient's general condition deteriorated. The day before the patient died, histopathological analysis showed prostatic abscess probably due to malignant lymphoma of the prostate and incidental adenocarcinoma. This is the first report of prostatic abscess with malignant lymphoma involving the prostate.

## 1. Introduction

Prostatic abscess is an uncommon urological disease but has a high mortality rate. In addition, prostatic lymphoma is rare. Here, we report an uncommon case of prostatic abscess due to malignant lymphoma involving the prostate.

## 2. Case Presentation

An 82-year-old man with a one-week history of urinary frequency and discomfort on urination was referred to our hospital in April 2014. The patient had a past history of total knee arthroplasty in 2009 but had no history of urological disease, including prostatic disease or any other type of disease prior to this illness, and had no previous complaints regarding urination. The patient was not taking any medications. Initial laboratory findings indicated microscopic hematuria and pyuria on urinalysis.* Klebsiella oxytoca* was detected on urine culture. Analysis of serum tumor markers revealed a serum prostate-specific antigen level of 2.56 ng/mL (normal range < 4 ng/mL). Digital rectal examination (DRE) revealed a soft and swollen prostate. A diagnosis of acute prostatitis was made and cefcapene was prescribed for 1 week.

**Figure 1:**
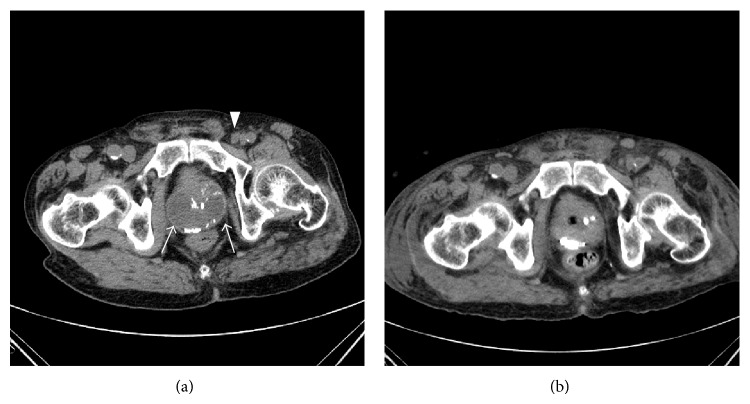
(a) CT revealed low-attenuating, round, well-demarcated fluid collection with calcification at the dorsal site of the prostate (arrows) and some lymph nodes were swollen near the prostate (arrowhead). The cystic lesion volume was about 50 mL. (b) CT revealed clear reduction of fluid collection at the dorsal site of the prostate.

**Figure 2:**
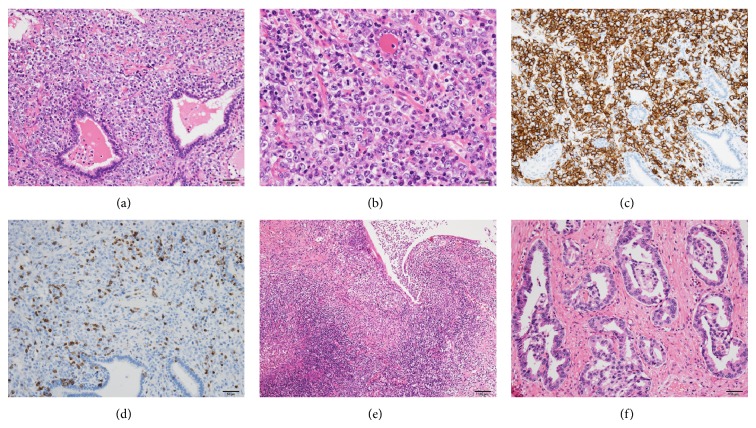
Histopathological examination. (a, b) Hematoxylin-eosin-stained section showing extensive infiltration of large B cells ((a) ×200, (b) ×400). (c, d) Immunohistochemistry panels showing positivity for CD20 ((c) ×200) and negativity for CD3 ((d) ×200). (e) Hematoxylin-eosin-stained section showing intercellular infiltration of inflammatory cells (×100). (f) Hematoxylin-eosin-stained section showing adenocarcinoma (×200). The Gleason score of the prostate cancer was 3 + 4.

The patient returned to our hospital in May 2014 with a two-day history of intermittent fever. The patient had no fever at the first visit, but on the second visit he showed a high fever of 38.9°C. On admission, computed tomography (CT) revealed a cystic lesion with calcification on the dorsal site of the prostate and some swollen lymph nodes near the prostate (Figure 1(a)). Laboratory findings revealed a white blood cell count of 7700/*µ*L without atypical cells, hemoglobin concentration of 9.2 g/dL, platelet count of 7.1 × 10^4^/*µ*L, and lactate dehydrogenase level of 440 IU/L (normal range 120–230 IU/L). The patient was initially treated with ceftazime. On hospital day 4, the intermittent fever remained and laboratory and physical findings indicated disseminated intravascular coagulation (DIC). Cystoscopy revealed no particular changes in the bladder or urethra. However, thick pus was milked out from the extrameatus by prostatic massage. Therefore, a clinical diagnosis of prostatic abscess was made. For drainage, we performed transurethral resection of the prostate (TURP) on hospital day 5.* Klebsiella oxytoca* was detected in the pus. From postoperative day 1, the patient was treated with thrombomodulin alfa (130 U/kg) for 5 days, immune globulin (2500 mg/body) for 3 days, and imipenem/cilastatin for 10 days. On postoperative day 5, CT showed clear reduction of prostatic abscess (Figure 1(b)). However, the patient's condition deteriorated. On postoperative day 12, histopathological findings of TURP specimens indicated diffuse proliferation of large cells with irregular nuclei and intercellular infiltration of inflammatory cells. Immunohistochemical analysis revealed positivity for CD20 and negativity for CD3. The pathological diagnosis was diffuse large B-cell lymphoma (DLBCL) and partly incidental adenocarcinoma (Figures 2(a)–2(f)). The patient died on postoperative day 13.

## 3. Discussion

Prostatic abscess is uncommon and its diagnosis is rare. The majority of cases of prostatic abscess present during the fifth or sixth decade of life and constitute approximately 0.5% of those hospitalized for prostatic disorders [1]. Diabetes mellitus, immunocompromised status, renal failure on prolonged dialysis, bladder outlet obstruction, recent prostatic biopsy, and prolonged catheterization are common predisposing factors in these cases [1]. This patient did not have a past history of diabetes mellitus, but it was possible that malignant lymphoma of the prostate was related to bladder outlet obstruction. The signs and symptoms of prostatic abscess are fever, urinary frequency disturbance, acute urinary retention, dysuria, perineal or lower back pain, and hematuria [2]. Prostatic abscess is difficult to diagnose, as it may mimic several other diseases of the lower urinary tract [3]. Prostatic abscess can progress to sepsis and death if not adequately treated or if treatment is delayed, and the data suggest a mortality rate ranging from 3% to 16% [4].* Escherichia coli* has the highest prevalence, occurring in about 70% of cases [5]. Accurate diagnosis and efficient treatment are therefore required [2]. However, no standard diagnostic or therapeutic methods have yet been established for prostatic abscess, because most data published regarding this condition are in the form of case reports [6].

Prostatic imaging studies such as transrectal ultrasonography (TRUS) and CT are important in diagnosis and treatment. TRUS can be used initially to easily make a diagnosis of prostatic abscess. The most common TRUS findings are one or more hypoechoic areas with well-defined and thick walls containing thick fluid [7]. However, the TRUS findings can be interpreted variously as other conditions, such as neoplastic processes, cystic lesions, or granulomas. Thus, in the initial stages of abscess formation, the results may be inconclusive. TRUS can also cause pain in patients, and abscess size may change with the angle of TRUS. CT of the abdomen and pelvic area causes no pain, is less subject to the viewpoint of the observer, and can help to detect contiguous spread of infection in nearby organs. The appearance of low-attenuating, round, well-demarcated fluid collections within the prostate gland by CT is suggestive of prostatic abscess [8]. We did not perform TRUS in this patient, because a clinical diagnosis of prostatic abscess was made earlier than by CT and DRE and did not select drainage by transperineal needle aspiration with TRUS.

Ludwig et al. [9] reviewed a series of 18 patients and suggested that a monofocal abscess <1 cm in diameter should be treated with intravenous broad-spectrum antibiotic therapy and a suprapubic catheter. Surgical drainage should be performed for multifocal abscesses >1 cm in diameter, septic shock, recurrent abscess, or patients responding poorly to antibiotics for 3 days or longer. Historically, the treatment of prostatic abscess included surgical interventions, such as TURP. Now, however, minimally invasive treatment, such as TRUS-guided needle aspiration or drainage via a tube transperineally or transrectally under local anesthesia or sedation, is preferred. However, there have been few reports comparing the treatment outcomes of prostate abscess according to the different modalities. Our patient suffered from DIC, so we carefully considered which methods should be used for drainage of the prostatic abscess, that is, transperineal needle aspiration, transgluteal drainage, or transurethral incision and resection of the prostate. We chose transurethral incision and resection of the prostate because the abscess volume was relatively large. The Foley catheter was put in place after drainage.

Lymphomas presenting at extranodal sites with or without slight lymph node infiltration are considered primary extranodal disease. Primary prostatic lymphoma is extremely rare, representing 0.1% of newly diagnosed lymphomas, and accounts for <0.09% of all prostate neoplasms [10]. In addition, with regard to the involved organ, lymphoma of the prostate is rare with either primary or even secondary involvement. This is mainly due to the lack of lymphoid tissue in this location, but the recognition of extramedullary hematopoiesis in the prostate confirmed the existence of lymphoma primary to the prostate [11]. The diagnostic criteria for primary lymphoma of the prostate have been proposed and included symptoms attributable to prostatic enlargement, lymphoma adjacent tissue, and lack of liver, spleen, lymph node, and peripheral blood involvement within 1 month of diagnosis [12]. In our case, we made a diagnosis of probable secondary lymphoma of the prostate, because some lymph nodes were swollen near the prostate.

The analysis included primary and secondary prostatic lymphoma, and lymphoma-specific survival was previously 33% at 5 years [12]. In our case, it was difficult to give the patient chemotherapy, because he had suffered from DIC until we obtained the histopathological diagnosis. However, a good prognosis has recently been reported for cases of primary prostatic lymphoma treated with chemotherapy alone or chemoradiotherapy [13–15]. As the R-CHOP (rituximab, cyclophosphamide, hydroxy daunorubicin, vincristine, and prednisone) regimen had become the new standard for nodal DLBCL, as in our case, some patients have been treated successfully with rituximab-combined chemotherapy [16, 17]. If the patient had visited our hospital in the early stages of the disease, the outcome would likely have been better.

## 4. Conclusions

We treated a patient who had prostatic abscess probably due to malignant lymphoma involving the prostate.
